# Amplitude of low-frequency fluctuation (ALFF) may be associated with cognitive impairment in schizophrenia: a correlation study

**DOI:** 10.1186/s12888-018-1992-4

**Published:** 2019-01-17

**Authors:** Pengshuo Wang, Jian Yang, Zhiyang Yin, Jia Duan, Ran Zhang, Jiaze Sun, Yixiao Xu, Luyu Liu, Xuemei Chen, Huizi Li, Jiahui Kang, Yue Zhu, Xin Deng, Miao Chang, Shengnan Wei, Yifang Zhou, Xiaowei Jiang, Fei Wang, Yanqing Tang

**Affiliations:** 1 Department of Psychiatry, The First Affiliated Hospital of China Medical University, Shenyang, Liaoning 110001 People’s Republic of China; 2 Brain Function Research Section, The First Affiliated Hospital of China Medical University, Shenyang, Liaoning 110001 People’s Republic of China; 3 Department of Gerontology, The First Affiliated Hospital of China Medical University, 155 Nanjing North Street, Heping District, Shenyang, Liaoning 110001 People’s Republic of China; 4 Department of Radiology, The First Affiliated Hospital of China Medical University, 155 Nanjing North Street, Heping District, Shenyang, Liaoning 110001 People’s Republic of China

**Keywords:** Schizophrenia, MCCB, ALFF

## Abstract

**Background:**

Cognitive impairments are prominent in schizophrenia (SZ). Imaging studies have demonstrated that functional changes of several areas of the brain exist in SZ patients. The relationships between these two indexes are largely unexplored in SZ. The MATRICS Consensus Cognitive Battery (MCCB) was used to measure cognitive impairment in multi-dimensional cognitive fields of SZ patients. This study was conducted to explore the relationship between cognitive functional impairment and the amplitude of low-frequency fluctuation (ALFF) in SZ patients.

**Method:**

A total of 104 participants (44 SZ patients and 60 age- and gender-matched healthy controls (HC)) were recruited for this study. The MCCB was used to assess cognitive function of the participants, while brain activity was assessed using the ALFF. The relationship between the MCCB and the ALFF was investigated by using a correlation analysis.

**Results:**

There were significant differences between SZ patients and HC in MCCB total and domain scores as well as in ALFF results. The reduction of ALFF in the bilateral postcentral gyri and paracentral lobule in SZ patients has a negative correlation with the MCCB sub-test of symbol coding.

**Conclusion:**

These findings suggest that the reduction of ALFF in bilateral postcentral gyri and paracentral lobule may be related to cognitive impairment in SZ patients.

**Electronic supplementary material:**

The online version of this article (10.1186/s12888-018-1992-4) contains supplementary material, which is available to authorized users.

## Background

The etiology of schizophrenia (SZ) as a mental disorder remains unclear. SZ has a heterogeneous clinical manifestation, and cognitive impairment presents as a common, prominent symptom [1–3]. Several studies have reported multiple cognitive impairments in SZ patients, including deficiencies in working memory, executive function, attention, processing speed, and social cognition [4, 5]. Additional areas of cognitive function, such as theory of mind [6] and autobiographical memory [7], may also be affected. The assessment of cognitive impairment is extremely important, as it may impact the processes of diagnosis, therapy, and rehabilitation [8].

The US National Institute of Mental Health (NIMH) developed the Measurement and Treatment Research to Improve Cognition in Schizophrenia (MATRICS) initiative to stimulate the development of new drugs that address the cognitive deficits associated with schizophrenia [9]. To accomplish this goal, the MATRICS consensus cognitive battery (MCCB) was developed, and it is currently widely used [10, 11]. The measurement of the cognitive impairment of SZ patients is estimated based on test–retest reliability, high utility as a repeated measure, relationship to functional outcome, potential changes in response to pharmacological agents, tolerability, and practicality [9, 12]. Based on previous research and input from experts, seven cognitive domains, including working memory, speed of processing, social cognition, attention-vigilance, verbal learning, visual learning, reasoning and problem-solving, were divided into 10 subtests. Several studies reported significant impairment in each of the seven MCCB domains in SZ patients compared to healthy controls (HC) [13–15]. After the MCCB was translated into Chinese, the clinical reliability and validity of MCCB were established between SZ patients and HC [16] to allow for its widespread use in cognitive research in SZ patients in China [17–19].

The resting state functional magnetic resonance imaging (R-fMRI) technique is a noninvasive and advanced neuroimaging measurement that has been recently used to investigate the pathophysiology of several disorders. The amplitude of low-frequency fluctuation (ALFF) [20] is an R-fMRI indicator that is used to detect the regional intensity of spontaneous fluctuations in the BOLD signal, which pinpoints the spontaneous neural activity of specific regions and physiological states of the brain. Electrophysiological studies [21] have shown that low-frequency oscillations may arise from spontaneous neuronal activity, which is of physiological significance and manifests in the rhythmic activity of the brain region through the interaction of information between connected brain regions; therefore, ALFF may reflect the characteristics of the brain [22]. ALFF studies suggested that intrinsic resting-state activity promotes or allows specific brain circuits to participate in cognitive tasks, and resting activity predicts subsequent task-induced brain responses and behavioral performance [23–25]. ALFF measures brain activity without cognitive load, and brain abnormalities in this state that correlate with cognition may be the basis of SZ cognitive impairment; therefore, we used ALFF in R-fMRI to examine SZ patients. Recent studies have reported gray matter ALFF changes in SZ patients. For example, Hoptman et al. [26] demonstrated that SZ patients, compared to HC, exhibited lower ALFF in the cuneus/precuneus, precentral gyrus, and lingual gyrus and exhibited higher ALFF in the left hippocampus/parahippocampus. A meta-analysis reported that SZ patients showed decreased ALFF in the bilateral occipital, posterior parietal cortices, sensorimotor cortex, and right superior temporal gyrus and elevated ALFF in the bilateral striatum, medial temporal cortex, medial prefrontal cortex, and lateral orbitofrontal cortex compared to that of HC [27]. Alonso-Solís et al. [28] revealed that SZ patients with auditory verbal hallucinations showed increased ALFF values in the bilateral temporal pole and parahippocampal gyrus and decreased ALFF values in the occipital pole, lingual gyrus, precuneus, and cingulate cortex. Non-hallucinating SZ patients showed increased ALFF values in the temporal fusiform and parahippocampal gyrus and decreased ALFF in the occipital lobe and precuneus. Zheng et al. [29] found that adolescents with early-onset SZ showed significantly increased ALFF in the bilateral orbitofrontal cortex and significantly decreased ALFF in the ventral precuneus. Another study reported that a first-episode SZ group showed significantly decreased ALFF in the orbital/medial frontal lobe and significant increases in ALFF in the left and right putamen [30].

Several researchers reported that SZ was accompanied with an impairment in cognitive domains, and the ALFF values in multiple brain regions were altered compared to those of HC. To the best of our knowledge, no previous study has investigated the relationship between cognitive impairment and changes in ALFF values in SZ patients. Therefore, the present study examined whether cognitive impairments in SZ patients were associated with ALFF values, as assessed by the MCCB and by R-fMRI. We hypothesized that cognitive function tested with the MCCB would be associated with ALFF in the brain regions that show changes during schizophrenia; for instance, working memory and social cognition may be associated with ALFF in the prefrontal cortex and information processing speed may be associated with ALFF in the postcentral gyri and paracentral lobule.

## Materials and methods

### Participants

Forty four patients who met DSM-IV criteria (American Psychiatric Association, 2000.) were recruited from the Department of Psychiatry, Frist Affiliated Hospital of China Medical University and the inpatient department of Mental Health Center of Shenyang, during the study, 41 patients were treated with medication, while the remaining 3 were not. 60 HC were recruited by advertisement from the community. Inclusion criteria for the study are as follows: 1) must be between the ages of 16–45, must be diagnosed with SZ according to DSM-IV-TR standards (at least two of the following symptoms: hallucinations, delusions, disorganized speech and catatonic or bizarre behavior and negative symptoms; symptoms persist for at least 1 month and continuous signs more than 6 months; social function impairment; exclude schizoaffective /mood disorder and substance abuse), and the diagnoses must be confirmed by 2 trained psychiatrists using the Structured Clinical Interview for DSM-IV Axis I Disorders (SCID-I); 2) HC subjects must not have current or life time Axis I Disorders, nor could they have any first-degree relatives with a history of Axis I disorders. Exclusion criteria for the study are as follows: 1) must not have a history of major physical disorders, particularly those that may be associated with brain tissue changes such as hypertension, diabetes, or metastatic disease; 2) must not have: unstable diseases such as heavy asthma; neurological abnormalities, including major head trauma (loss of consciousness lasting more than 5 min), epilepsy, cerebrovascular disease, brain tumors, or neurodegenerative diseases; somatic diseases that may cause mood disorders such as multiple sclerosis, thyroid disease, etc.; 3) no MRI contraindication; and 4) no lifetime or current substance dependence or abuse. All participants signed informed consent as approved by the Ethics Committee of China Medical University.

### Cognitive assessment

Assessment of neurocognitive functioning was completed using the MATRICS Consensus Cognitive Battery (MCCB). This instrument includes 10 tasks across 7 cognitive domains, including: speed of processing (Brief Assessment of Cognition in Schizophrenia Symbol Coding, Category Fluency, Trails A), attention-vigilance (Continuous Performance Test), working memory (WMS-III Spatial Span, Letter Number Span), verbal learning (Hopkins Verbal Learning Test – Revised), visual learning (Brief Visuospatial Memory Test – Revised), reasoning and problem solving (The Mazes test), and social cognition (Mayer – Salovey – Caruso Emotional Intelligence Test). The MCCB yields 10 subscale scores, 7 domain scores, and a composite score, calculated across the 7 domains. The MCCB shows good test–retest reliability (with scores ranging from *r* = 0.69 to *r* = 0.85), practicality, and tolerability. The collective battery took approximately 60–90 min to administer. In this study, all patients with SZ and HC completed the MCCB.

### Image acquisition

Scanning took place on the 3 T MRI scanner (General Electric, Milwaukee, USA) at the Image Institute of First Affiliated Hospital of China Medical University, Shenyang, China. Earplugs and foam pads were used to minimize scanner noise and head motion. A standard head coil was used for radio frequency transmission and reception of the nuclear magnetic resonance signal. Functional images were collected using a gradient echo planar imaging (EPI) sequence (a 6 min 40 s resting state fMRI scan, TR = 2000 ms, TE = 40 ms, FOV = 24 cm × 24 cm, flip angle = 90°, matrix = 64 × 64, slices = 35, slice thickness = 3 mm, no gap). Participants were instructed to close their eyes, remain awake, and keep their mind blank during the resting state scan (after the scanning we checked this with the subjects).

### Image data processing

Image data processing was performed by using Data Processing Assistant for Resting-State fMRI (DPABI, 2.3, Advanced edition) [31]. We discarded the initial 10 scan volumes to allow for steady-state magnetization. Then corrected for slice timing and head motion (using a least squares approach and a six-parameter spatial transformation). We excluded participants whose head motion exceeded 3 mm or rotation that exceeded 2.5° during scanning. The standard Montreal Neurological Institute (MNI) template provided by SPM was then used for spatial normalization with a resampling voxel size of 3 mm × 3 mm × 3 mm. The images were spatially smoothed with a 6-mm full-width at half-maximum Gaussian filter. We conducted time series linear detrending and temporal band-pass filtering (0.01–0.08 Hz) to remove low-frequency drifts and physiological high-frequency noise.

The ALFF analysis was carried out using DPABI. The filtered time series of each voxel was transformed into the frequency domain with a Fast Fourier Transform and the power spectrum was then obtained. We were measured ALFF by obtaining the square root of the signal across 0.01–0.08 Hz for each voxel [20]. For standardization purposes and to reduce the influence of individual variation in ALFF values, the ALFF of each voxel was further divided by the global mean of ALFF values for each subject within the default brain mask from the DPABI, with background and other non-brain tissue signals removed. This created a standardized whole-brain ALFF map.

### Statistical analysis

We performed two-sample t-tests on MCCB scores between the two groups, and compared the differences in total score and each sub-test score, the significant level was set at *p* < 0.05. Two-sample t-tests were used to compare ALFF data between the SZ patients and HC using DPABI to found significant brain regions, the significant level was set at *p* < 0.001 (GRF, Gaussian random field corrected).

### Analysis of associations between ALFF and MCCB scores

To explore the detailed associations between ALFF and MCCB scores in time, the following analysis was conducted, ALFF values were extracted from the significant regions. The correlation coefficient between the significant ALFF values in significant regions and MCCB scores in SZ group was calculate using SPSS (Statistical Product and Service Solutions) 22.0 software (SPSS Inc., Chicago, Illinois) software, and results were corrected by False Discovery Rate (FDR) correction (q < 0.05).

## Results

### Demographic data outcome

There were no significant differences between SZ and HC groups in age (*p* = 0.250) or gender (*p* = 0.866), antipsychotic drug doses were converted to chlorpromazine equivalent [32]. The results of demographic data were listed in Table 1.

**Table 1:** Demographic data of all subjects

	SZ (*n* = 44)	HC (*n* = 60)	t/χ^2^	*p*
Age (years, mean ± S.D.)	25.00 ± 7.49	30.13 ± 8.49	−3.260	0.250
Sex (female/male)	31/13	37/23	0.352	0.866
Education (years, mean ± S.D.)	14.35 ± 3.73	12.52 ± 3.05	2.659	0.009
Medication (Y/N)	41/3	N/A.		
First episode (Y/N)	23/21	N/A.		
Duration (month, mean ± S.D.)^a^	43.02 ± 42.42	N/A.		
Medication-CPZ Equivalent	365.373 ± 150.8	N/A.		
BPRS (total, mean ± S.D.)^a^	27.25 ± 9.13	18.46 ± 1.57	7.315	< 0.001

Note: *SD* Standard deviation, *Y* Yes, *N* No, *N/A* None, *BPRS* Brief Psychiatric Rating Scale

^a^includes missing information for some participants

In the SZ group, 40 were taking atypical antipsychotics including clozapine (y = 0.6903x + 69.747), risperidone (y = 0.0116x + 0.0446), olanzapine (y = 0.0332x + 2.0093), aripiprazole (y = 0.0266x + 5.311), ziprasidone (y = 0.1649x + 46.134) and quetiapine (y = 0.9004x + 85.459) chlorpromazine is represented by “x” in the above formulas, 1 was taking paroxetine, and 3 were not taking any psychotropic medication at the time of scan. All antipsychotic doses were converted to chlorpromazine equivalents using standard procedures, added to demographic data

### ALFF group differences

**Fig. 1:**
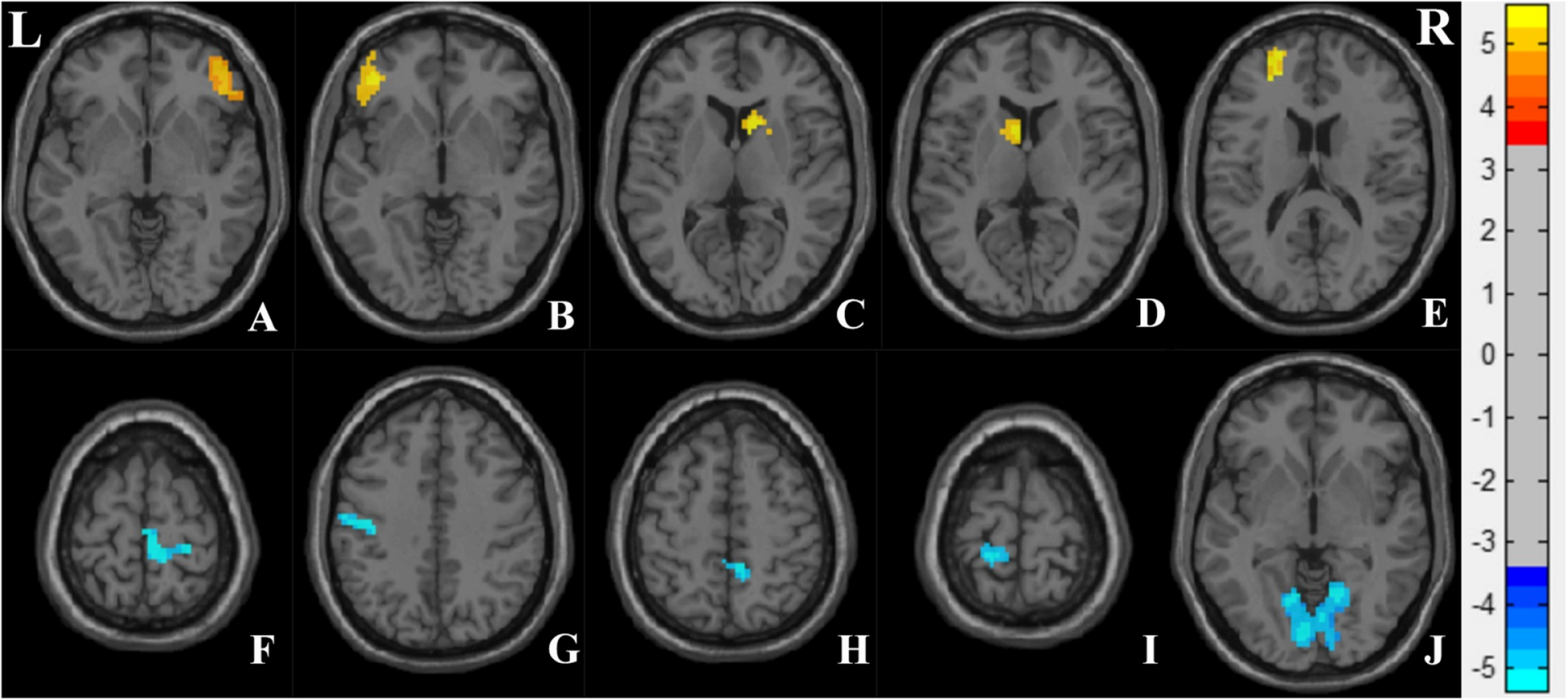
Regions showing altered amplitude of low frequency fluctuation (ALFF) in the SZ group, compared to the HC group. The color bar represents the range of t values. L, left; R, right

ALFF analysis yielded 10 clusters, that includes 12 regions. We used Anatomical Automatic Labeling template to identify the brain regions in the clusters using DPABI software. The SZ group showed significantly decreased ALFF in the bilateral lingual, calcarine, cuneus, postcentral, paracentral lobule, and right precuneus. Furthermore, the SZ group had significantly increased ALFF in the bilateral caudate, orbital part of the inferior frontal gyrus, middle frontal gyrus, medial superior frontal gyrus, triangular inferior frontal gyrus, and superior frontal gyrus (Fig. 1, Table 2), in the figure, the yellow area shows that the ALFF value of SZ is higher than HC, and the blue area shows that the ALFF value of SZ is lower than HC.

**Table 2:** Regions with ALFF differences in SZ and HC subjects

	Brain regions	Cluster size	Peak coordinates (MNI)			T values
			x	y	z	
SZ>HC	A L Medial superior frontal gyrus	237	51	27	6	5.6597
	R Middle frontal gyrus	151				
	R Medial superior frontal gyrus	127				
	R Superior frontal gyrus	123				
	R Pars triangularis inferior frontal gyrus	94				
	R Orbital inferior frontal gyrus	82				
	L Superior frontal gyrus	62				
	B L Pars triangularis inferior frontal gyrus	82	−39	42	−9	5.0682
	L Orbital inferior frontal gyrus	58				
	C R Caudate	75	12	12	6	5.0632
	D L Caudate	79	−9	9	3	5.0506
	E L Medial frontal gyrus	40	−24	54	18	4.4804
SZ<HC	F R Paracentral lobule	36	12	−36	69	−4.2799
	R Postcentral gyrus	24				
	G L Postcentral gyrus	136	−51	−12	33	−4.3341
	H R Precuneus	41	9	−48	51	−4.5002
	I L Postcentral gyrus	38	−18	−39	72	−4.9716
	L Paracentral lobule	23				
	J L Calcarine	200	6	−81	15	−5.3827
	R Lingual gyrus	183				
	R Calcarine	157				
	L Lingual gyrus	156				
	L Cuneus	76				
	R Cuneus	52				

Peak coordinates refer to the point with the highest t value in the cluster, not the specific region

Note: x, y, z coordinates of peak locations in the Montreal Neurological Institute space (MNI)

*ALFF* Amplitude of low frequency fluctuations, *HC* Healthy control, *SZ* Schizophrenia patients, *L* Left, *R* Right

### Cognitive assessment outcome

**Fig. 2:**
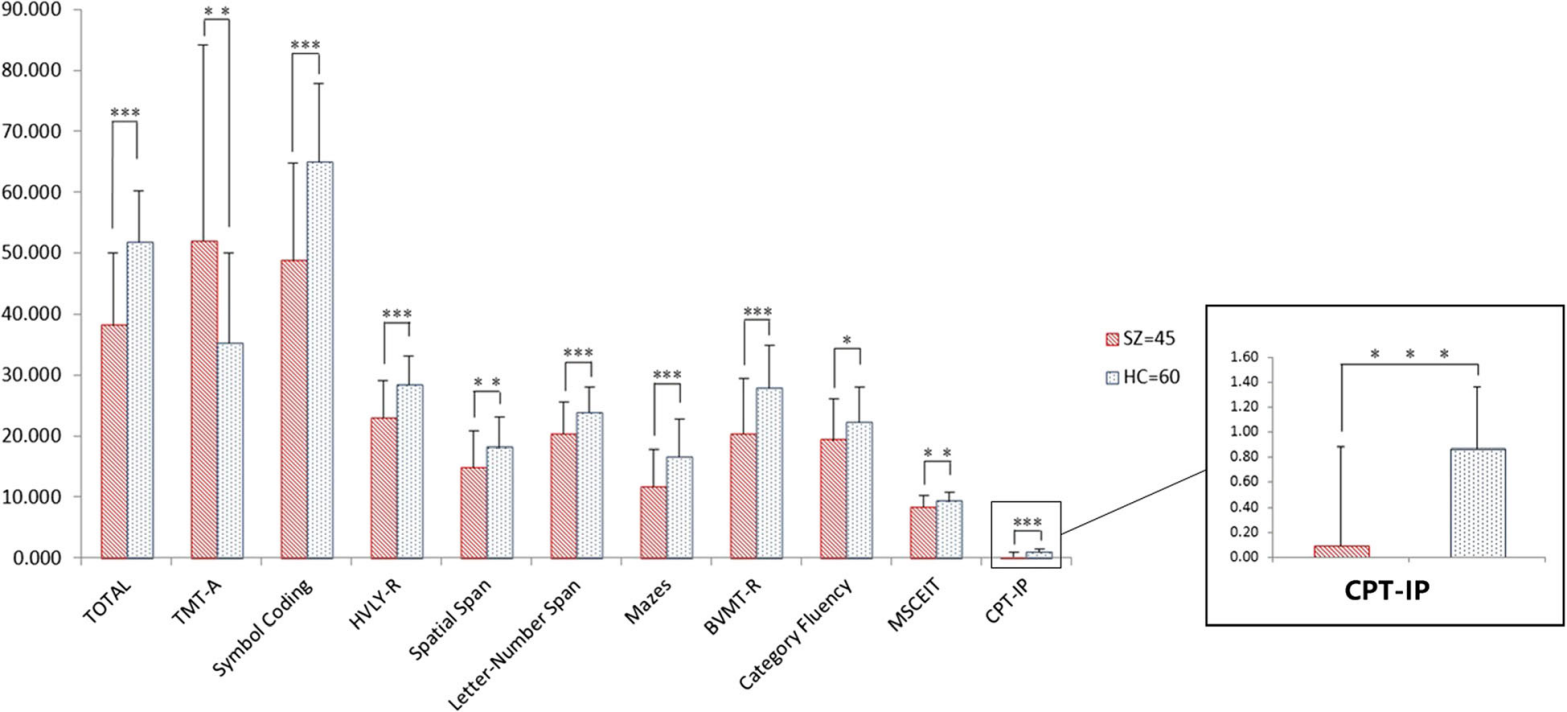
MCCB Scores by diagnosis. TMT-A = Trail Making Test A, HVLT-R = The revised Hopkins Verbal Learning Test, BVMT-R = The revised Brief Visuospatial Memory Test, MSCEIT = Mayer-Salovey-Caruso Emotional Intelligence Test, CPT-IP = The Continuous Performance Test Identical Pairs. Note: * *p* < 0.05, ** *p* < 0.01, *** *p* < 0.001

Compared to the HC group, the SZ group showed a significantly impaired total score and were impaired in all domains (Fig. 2).

### Correlations between ALFF and MCCB

**Fig. 3:**
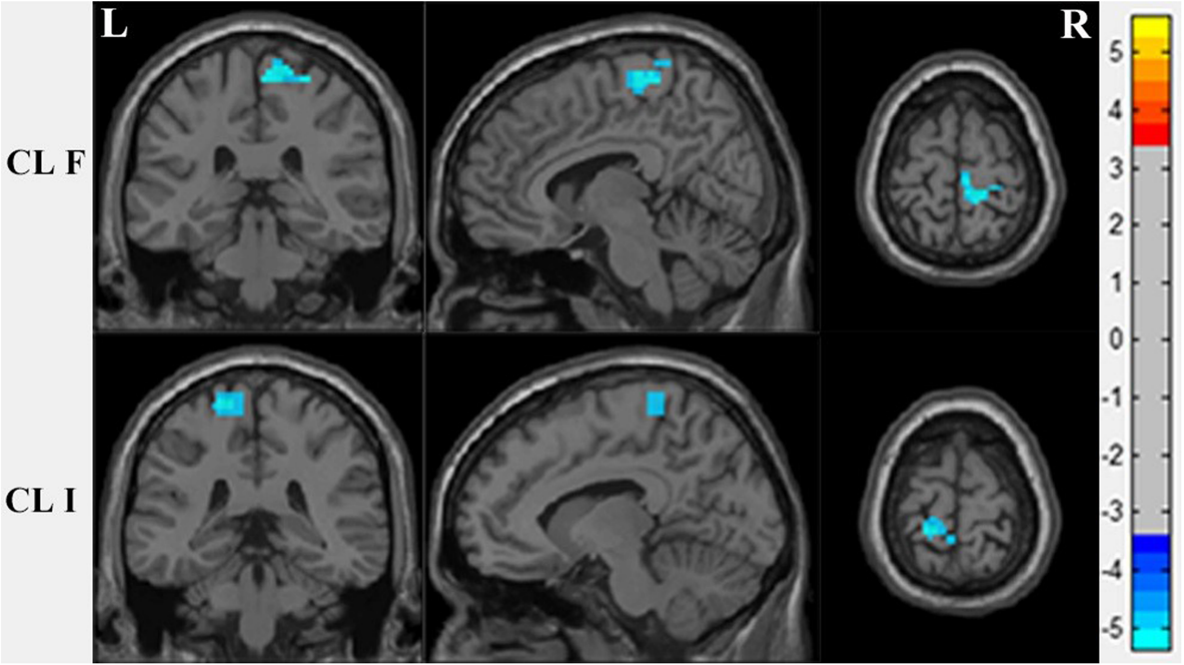
These regions associated with symbol coding, after the FDR correction (p < 0.05). The color bar represents the range of t values. L, left; R, right; CL, cluster

**Fig. 4:**
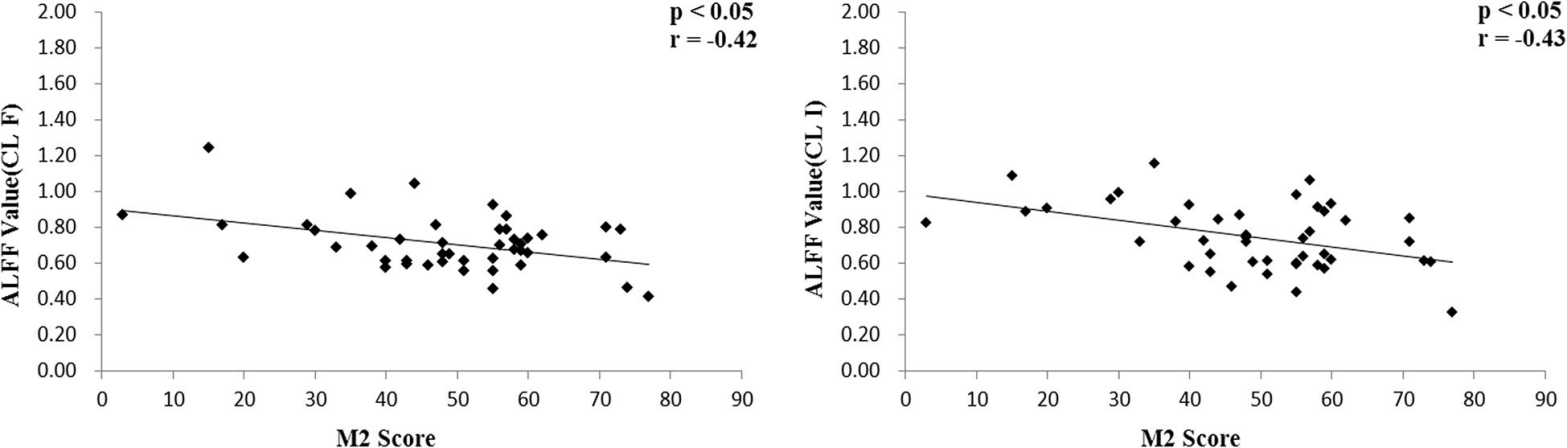
Scatter diagram describing the relevant trends of cluster F and I, ALFF value, and the symbol coding score

Both cluster F (Right paracentral lobule and postcentral gyrus) (*p* < 0.05, *r* = − 0.42) and cluster I (Left postcentral gyrus and paracentral lobule) (p < 0.05, *r* = − 0.43) have a negative correlation with the MCCB symbol coding sub-test (M2) in SZ patients, results after the FDR correction (q < 0.05) (Figs. 3 and 4). When we use stricter significance levels of FDR correction or Bonferroni correction the results cannot hold. We also computed correlations in healthy samples, and there was a potentially positive correlation existed between the two brain regions and MCCB data without corrections (Additional file 1).

## Discussion

The present study found cognitive impairments and ALFF differences between SZ patients and HC. Specifically, there was a negative correlation between the symbol-coding subtest and ALFF values in the bilateral postcentral gyri and the paracentral lobule in SZ patients.

We found that SZ patients showed cognitive impairments in all domains, which is consistent with previous studies that used the same cognitive measurement [15]. Deficits processing speed [33], working memory [34], verbal learning [7], reasoning/problem-solving [35], visual learning [36], and attention/vigilance [37] were found in SZ patients. Similar cognitive impairments were also reported in studies investigating patients with other psychiatric diagnoses [38–41]; however, the cognitive deficits were not as severe or as widespread as those seen in SZ patients. Therefore, the differences in the extent of cognitive impairment are likely due to the unique pathophysiological mechanisms of SZ. Our R-fMRI results showed increased ALFF in the bilateral caudate, the middle frontal gyri, and the left superior frontal gyrus, which is consistent with the established findings in SZ patients [42]. Tumbwene et al. [43] reported decreased ALFF changes similar to ours in the bilateral orbital inferior frontal gyri. A meta-analysis [27] also reported increased ALFF in the medial superior frontal gyri, which is consistent with our study. Furthermore, previous studies have reported decreased ALFF in the bilateral lingual [28], calcarine, cuneus, and postcentral gyri [44] areas of the brains of SZ patients (Table 3).

**Table 3:** Overlaps between our study and previous resting-state functional Magnetic Resonance Imaging studies

	Our study	Previous study
Increased ALFF	bilateral caudate	Turner et al. 2013
	middle frontal gyri	Turner et al. 2013
	left superior frontal gyrus	Turner et al. 2013
	medial superior frontal gyri.	Xu et al. 2015
Decreased ALFF	bilateral orbital inferior frontal gyri	Mwansisya et al. 2017
	bilateral lingual	Alonso-Solis et al. 2017
	bilateral calcarine	Salvador et al. 2017
	bilateral cuneus	Salvador et al. 2017
	bilateral postcentral gyri	Salvador et al. 2017
Each article reported different	hippocampus	Previous literature reports were inconsistent in these ragion.
	parahippocampus	
	sensorimotor cortex	
	temporal pole	
	parahippocampal gyrus	
	temporal fusiform	
No clear report	bilateral pars triangularis inferior frontal gyrus	

Previous studies reported ALFF changes in the hippocampus/parahippocampus [26], temporal pole, parahippocampal gyrus [28] and temporal fusiform [29], but these anomalies were not found in the present study; these differences may be due to population heterogeneity, different cultural backgrounds and different experimental parameters.

The symbol-coding subtest in the MCCB was used to evaluate processing speed in our study, which was also related to decreased ALFF in the bilateral postcentral gyri and paracentral lobule of SZ patients. These two areas are adjacent; the postcentral gyrus is located in the primary somatosensory cortex, and the paracentral lobule encompasses the medial continuation of the pre- and postcentral gyrus. The anterior region in the frontal lobe forms the supplementary motor area. Processing speed is important for SZ patients, and impairment in this area has been widely reported in previous studies [13, 15, 33]. Impairment in processing speed is an important factor for predicting the functional outcome of SZ patients [45–48]. Compared to other affected domains, processing speed exhibits the most serious deficit in SZ patients, with an average of approximately 1.5 standard deviations below that of HC [45]. The nature of the neuropsychological testing of processing speed requires participants to rapidly execute relatively simple tasks that integrate many basic cognitive operations. Therefore, it is not surprising that brain imaging studies on processing speed focused on reaction time. A previous task-state study reported [49] higher activity in the postcentral gyrus when subjects responded slower relative to previous responses. In the present study, activity in the postcentral gyrus in SZ patients was also negatively correlated with the speed of processing. Therefore, in this study, higher postcentral gyrus activity may lead to slower reaction times, which may impact processing speed in SZ patients. The somatosensory cortex is related to reaction times [50], and abnormalities in the postcentral gyrus, which is part of somatosensory cortex, may be the cause of longer reaction times in patients and may affect the speed of processing. Another region associated with processing speed is the bilateral paracentral lobule, which is part of the sensorimotor region. The paracentral lobules project to downstream motor nuclei and react to movements [51–54], which potentially contributes to motor function deficits in SZ patients and is associated with dysfunction and structural abnormalities in this region [55]. We also found potential positive correlations in healthy samples between these two brain regions and the symbol-coding subtest. However, these correlations disappeared with FDR correction (q < 0.05). A previous study on fractional ALFF and cognition (attention) also found opposite trends in SZ vs HC [56]. We speculate that this correlation may be a special functional pattern of the brain in SZ patients after cognitive impairment.

We also observed increased ALFF in the bilateral pars triangularis inferior frontal gyrus of SZ patients, which has not been reported previously. The pars triangularis is part of Broca’s region and plays a role in language and interpersonal information processing. The pars triangularis inferior frontal gyrus is related to the pathogenesis of positive symptoms, and gray matter volume reductions in this area may represent a vulnerability to SZ, which is considered to be a way to determine the developmental trajectory of disease [57]. However, the neurobiological basis for our finding remains unclear. Although there was no relationship between this area and the MCCB subtest in the present study, the significance of the anomalies in this region require further investigation.

There are some limitations in our research. Participants in the resting-state MRI study were required not to think during the scan, but this is an ideal state. Thinking may have affected the results of the experiment. This factor is a problem that remains unsolved. Increasing the sample size, retesting the same sample and measuring the stability of the index may be future solutions. We were unable to control the medications prescribed to our participants, thus some patients received medical treatment, while others did not; therefore, the effects of antipsychotics on cognitive function and ALFF cannot be discounted. Future studies should recruit people with schizophrenia who are initially untreated. Additionally, some data from the duration and BPRS (Brief Psychiatric Rating Scale) scores were missing despite our best efforts to collect all data; thus, our findings should be cautiously interpreted due to insufficient data from some participants. Our correlation results did not withstand stricter FDR significance levels or Bonferroni correction (*p* < 0.05). We cannot verify results on public SZ datasets because of the lack of fMRI and MCCB data.

## Conclusion

Our results support the hypothesis of an underlying relationship between ALFF and cognition impairment (e.g., speed of processing) in SZ patients. In the present study, SZ patients displayed decreased ALFF in the bilateral postcentral gyri and paracentral lobule, and both areas may be associated with the symbol-coding subtest in SZ patients.

## Additional file

Additional file 1: Supplementary materials. (DOCX 250 kb)

## Abbreviations

ALFF: Amplitude of low-frequency fluctuation
BPRS: Brief Psychiatric Rating Scale
EPI: Echo planar imaging; MNI: Montreal Neurological Institute
FDR: False Discovery Rate
FFT: Fast Fourier Transform
GRF: Gaussian random field
HC: Healthy controls
MCCB: MATRICS consensus cognitive battery
R-fMRI: Resting state functional magnetic resonance imaging
SCID-I: Structured Clinical Interview for DSM-IV Axis I Disorders
SD: Standard deviation
SPSS: Statistical Product and Service Solutions
SZ: Schizophrenia
