# Supplementary material for: Amplitude of low-frequency fluctuation (ALFF) may be associated with cognitive impairment in schizophrenia: a correlation study
Source: BMC Psychiatry. 2019 Jan 17;19:30. doi: 10.1186/s12888-018-1992-4 (PMC6337807; doi:10.1186/s12888-018-1992-4)

Education data have been added the original text, and we conducted image analysis with education as the covariate, the difference between the two groups is consistent with previous results. Socioeconomic, vocational status, etc. were not collected, but we may investigate these further in future studies.


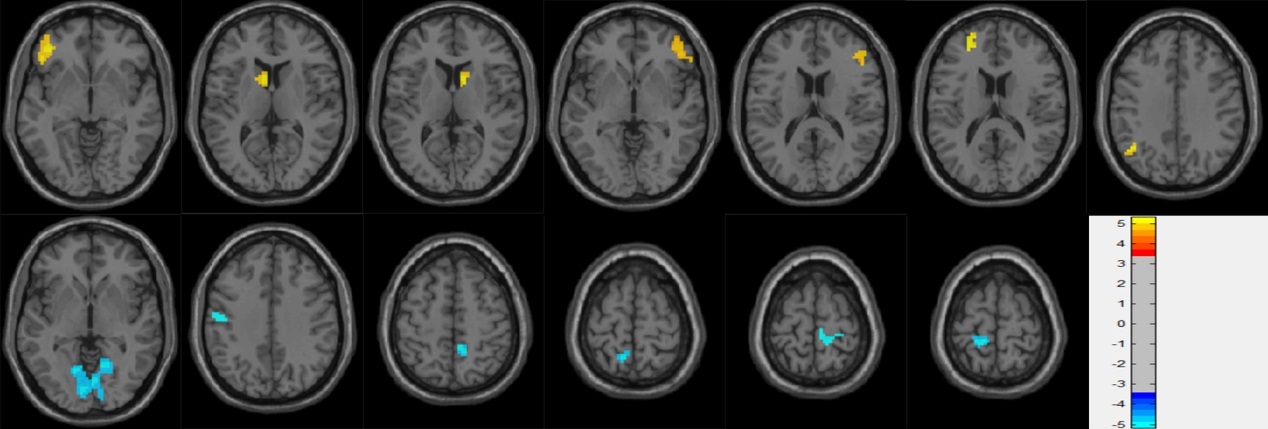


We also discussed correlations in healthy samples, but no correlation existed between the two brain regions and MCCB data after corrections. Based on our aims, we just wanted to explore the correlation between brain function and cognitive impairment in patients with schizophrenia; thus, the results of healthy people were not included in this article.


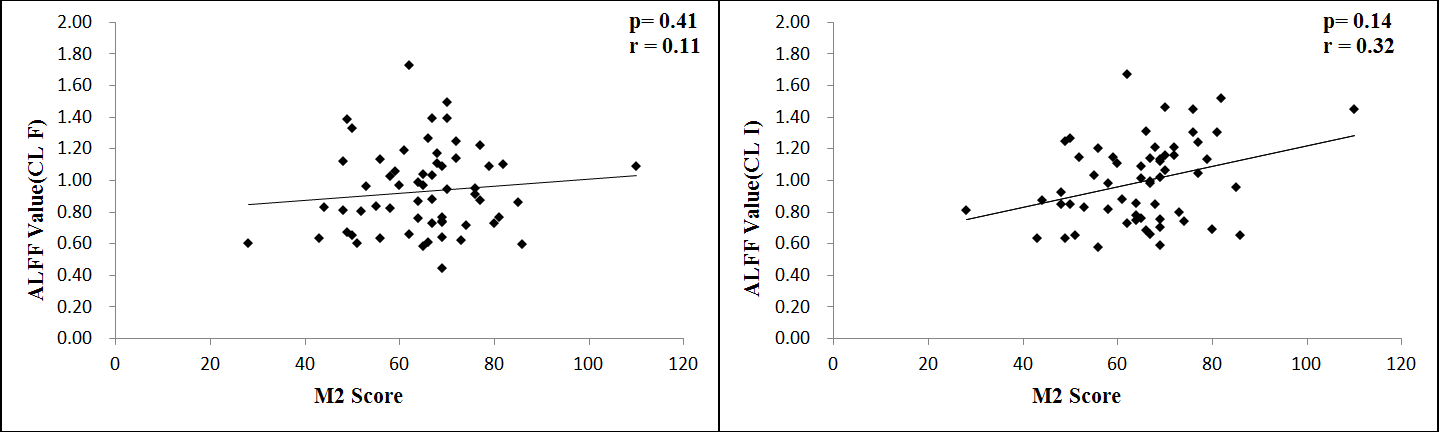


The two clusters fuse together when *p* = 0.005; correlation still occurs, *p* < 0.05, r = -0.483. A correlation between the two clusters was present (I & J), *p* < 0.05, r =0.731.


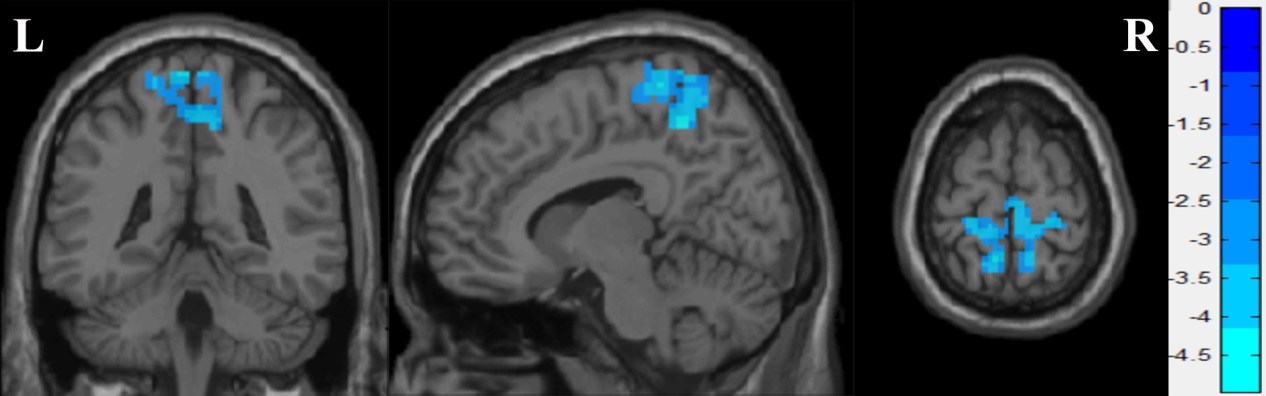

Supplement: Supplementary file 1 — Supplementary materials. (DOCX 250 kb) [file 12888_2018_1992_MOESM1_ESM.docx]
